# Can exercise therapy combined with transcranial direct current stimulation further improve balance ability in individuals with chronic ankle instability? A systematic review and meta-analysis

**DOI:** 10.3389/fphys.2025.1681272

**Published:** 2025-10-15

**Authors:** Jiawei Zheng, Ruixiong Chen, Sifan Wang, Jian Chen, Xikai Lin

**Affiliations:** ^1^ Engineering Research Center of Sports Health Intelligent Equipment of Hubei Province, Wuhan Sports University, Wuhan, Hubei, China; ^2^ Department of Rehabilitation Medicine, The Third Affiliated Hospital, Sun Yat-sen University, Guangzhou, Guangdong, China; ^3^ Beijing University of Chinese Medicine Shenzhen Hospital (Long gang), Shenzhen, Guangdong, China

**Keywords:** transcranial direct current stimulation, chronic ankle instability, sports therapy, dynamic balance ability, static balance ability

## Abstract

**Objectives:**

To evaluate whether transcranial direct current stimulation (tDCS) combined with exercise therapy enhances balance ability in chronic ankle instability (CAI) individuals.

**Methods:**

Systematic searches were conducted in PubMed, Embase, Web of Science, Cochrane Library, and Scopus databases up to July 10, 2025. The Cochrane Risk of Bias tool version 2. Was used to assess the methodological quality of studies meeting the inclusion criteria. Meta-analyses were performed using random-effects models, with results expressed as standardized mean differences (SMD) and 95% confidence intervals (95% CI). Evidence quality was evaluated using the GRADE methodology.

**Results:**

Eight studies involving 216 participants were included in the meta-analysis. Overall analysis revealed that tDCS combined with exercise therapy did not significantly improve dynamic balance (SMD: 0.08, 95% CI: −0.36 to 0.52, *P* = 0.72) or static balance (SMD: −0.53, 95% CI: −1.08 to 0.02, *P* = 0.06) in individuals with CAI. Subgroup analysis by exercise type showed that tDCS combined with non-balance training significantly enhanced dynamic balance ability (SMD: 0.52, 95% CI: 0.07 to 0.97, *P* = 0.02), while tDCS combined with balance training showed no significant improvements in either composite dynamic balance measures (SMD: −0.26, 95% CI: −0.82 to 0.30, *P* = 0.36) or Y-balance reach distances in any direction (*P* > 0.05 for all directions).

**Conclusion:**

tDCS provides therapeutic benefits for dynamic balance only when combined with non-balance exercises. Current evidence is insufficient to demonstrate improvements in static balance with tDCS adjunctive therapy.

## 1 Introduction

Lateral ankle sprains are among the most common types of sports injuries, with approximately 70% of first-time sprain patients eventually developing chronic ankle instability (CAI) (Herzog et al., 2019). Individuals with CAI frequently experience symptoms such as recurrent ankle “giving way,” repeated sprains, functional limitations, and diminished balance ability, which significantly impact daily activities and athletic performance (Gribble et al., 2016; Thompson et al., 2016). Extensive research has confirmed that exercise therapy can improve balance control and reduce re-injury risk in individuals with CAI by enhancing ankle proprioception and ankle muscle strength, making it the preferred conservative treatment approach for CAI (Yang et al., 2025; Zhang et al., 2025a). Recent studies have revealed that CAI development is not solely related to local ligament and proprioceptive damage, but also involves adaptive changes within the central nervous system (Maricot et al., 2023; Li et al., 2025). These changes are specifically characterized by decreased neural excitability at the spinal and/or cortical levels, which subsequently leads to altered muscle activation patterns in the lower extremity and compensatory biomechanical adaptations (Suttmiller and McCann, 2020; Li et al., 2025). However, traditional exercise therapy has certain limitations in promoting central nervous system remodeling and improving cortical excitability.

As a non-invasive neuromodulation technique, transcranial direct current stimulation (tDCS) employs weak direct electrical currents delivered between electrodes placed on the cerebral cortex. This technique modulates cortical excitability by altering neuronal resting membrane potentials, thereby promoting neuroplasticity and enhancing motor learning capacity (Auvichayapat and Auvichayapat, 2011; Chase et al., 2020). In rehabilitation of neurological conditions such as Parkinson’s disease, tDCS has been proven effective as an adjunct to conventional rehabilitation, further enhancing patients’ balance and postural stability (Nguyen et al., 2024). For individuals with CAI, researchers have attempted to combine exercise therapy with tDCS to explore the comprehensive therapeutic effects. However, current research findings remain inconsistent, Ma et al. found that combining tDCS with short-foot exercises improved dynamic balance ability in CAI individuals compared to short-foot exercises alone (Ma et al., 2020), while Kim et al. revealed that tDCS combined with active joint mobilization training did not provide significant additional clinical benefits (Kim et al., 2025).

Given the inconsistent research results regarding tDCS combined with exercise therapy for CAI treatment and the lack of systematic synthesis, this study aims to comprehensively evaluate the therapeutic efficacy of tDCS combined with exercise therapy *versus* exercise therapy alone in improving balance ability in CAI individuals using systematic review and meta-analysis methodology. By integrating existing research evidence, we seek to determine whether tDCS can serve as an effective adjunctive treatment to further enhance patients’ balance ability, while analyzing key factors that may influence treatment outcomes to provide reliable evidence-based guidance for clinical practice.

## 2 Materials and methods

This study adhered rigorously to the Preferred Reporting Items for Systematic Reviews and Meta-Analyses (PRISMA) guidelines and the Cochrane Handbook (Cumpston et al., 2019; Page et al., 2021). The study protocol has been registered on the PROSPERO platform (CRD420251110630).

### 2.1 Search strategy

Two researchers (ZJW and CRX) independently conducted searches in PubMed, Embase, Web of Science, Cochrane Library, and Scopus databases. The search was conducted up to July 10th. The specific search strategies for each database are provided in Supplementary Table S1.

### 2.2 Eligibility criteria

Inclusion criteria: 1) Participants were individuals with CAI as defined by the Intentional Ankle Consortium statement; 2) The intervention group received tDCS combined with any form of exercise therapy, while the control group underwent exercise therapy alone or exercise therapy supplemented with sham tDCS intervention; 3) Outcome measures included balance ability assessments; 4) Studies were randomized controlled trials. Exclusion criteria: 1) Publications in languages other than English; 2) Animal studies and conference abstracts.

### 2.3 Study selection and data extraction

Screening of the retrieved studies was conducted according to predetermined inclusion and exclusion criteria through a systematic examination of study titles, abstracts, and complete manuscripts. The following basic characteristics were extracted from studies that satisfied the final criteria: 1. First author, country, and publication year; 2. Number of participants, gender, and age; 3. Intervention methods for experimental and control groups, including intervention duration and frequency; 4. Outcome measures and measurement timepoints; 5. tDCS electrode placement, stimulation intensity, stimulation duration, sham tDCS protocol, and adverse events; 6. Changes in balance assessment parameters following intervention were measured using multiple indicators: the dynamic postural stability indices (DPSI) and Y balance test (YBT) for evaluating dynamic balance capabilities, and center of pressure (COP) displacement and balance error scoring system (BESS) for assessing static balance performance. Two researchers (ZJW, CRX) performed all operations independently, with conflicts resolved through consensus discussion involving a third reviewer (WSF).

When studies did not provide complete data, emails were sent to corresponding authors to request the missing information. For studies presenting data in graphical format, data extraction was performed using GetData software (V2.26).

### 2.4 Bias risk assessment

Two researchers (ZJW, CRX) independently assessed the risk of bias for each included study using the Cochrane Risk of Bias tool version 2 (RoB 2) (Sterne et al., 2019). This assessment tool evaluates five critical domains: randomization process, deviations from intended interventions, missing outcome data, measurement of outcomes, and selection of reported results. Each domain was rated as low risk, some concerns, or high risk of bias. Any disagreements between the two assessors were resolved through consultation with a third researcher (WSF).

### 2.5 Certainty of evidence

Two independent researchers (ZJW, CRX) conducted evidence quality assessment of the meta-analysis results using GRADEpro software. Five domains were evaluated: bias risk, inconsistency, indirectness, imprecision, and publication bias, leading to evidence quality ratings of high, moderate, low, or very low (Guyatt et al., 2011). A third researcher (WSF) resolved any assessment disagreements.

### 2.6 Statistical analysis

Review Manager V.5.3 software was utilized to perform the meta-analysis, analyzing the mean and standard deviation (SD) of baseline-to-post-intervention changes for both the intervention and control groups in each study. When required data were unavailable in the original publications, calculations were undertaken using methods derived from previous research approaches (Ye et al., 2023). Due to variations in intervention protocols and measurement units among the included studies, all outcome measures were synthesized using a random-effects model and expressed as standardized mean differences (SMD) with 95% confidence intervals (CI) (Borenstein et al., 2010; Andrade, 2020). Specifically, Hedges’ g was used to calculate the SMD, with effect sizes interpreted as follows: >0.8 indicating a large effect, 0.5–0.8 a medium effect, 0.2–0.5 a small effect, and <0.2 a trivial effect (Wu et al., 2021). The I^2^ statistic assessed between-study heterogeneity: I^2^ < 30% (no heterogeneity), 30%–50% (moderate heterogeneity), 50%–75% (substantial heterogeneity), and >75% (considerable heterogeneity) (Cumpston et al., 2019). To examine result stability, sensitivity analysis was conducted by excluding studies individually and assessing how these removals affected the calculated effect sizes and their statistical significance (Viechtbauer and Cheung, 2010). Statistical significance was defined as *P* < 0.05 for the overall effect.

Given that balance training has been proven to significantly improve balance function in individuals with CAI (Guo et al., 2024), subgroup analyses were further conducted based on the type of exercise intervention to explore the differential effects of tDCS combined with different exercise modalities on balance ability in CAI, specifically categorized into tDCS combined with balance training group and tDCS combined with non-balance training group. Additionally, given that the YBT involves three testing directions, subgroup analyses were also performed based on YBT directions (anterior, posteromedial, and posterolateral) to further evaluate the direction-specific effects of tDCS on dynamic balance ability in individuals with CAI.

## 3 Results

### 3.1 Study selection

**FIGURE 1:**
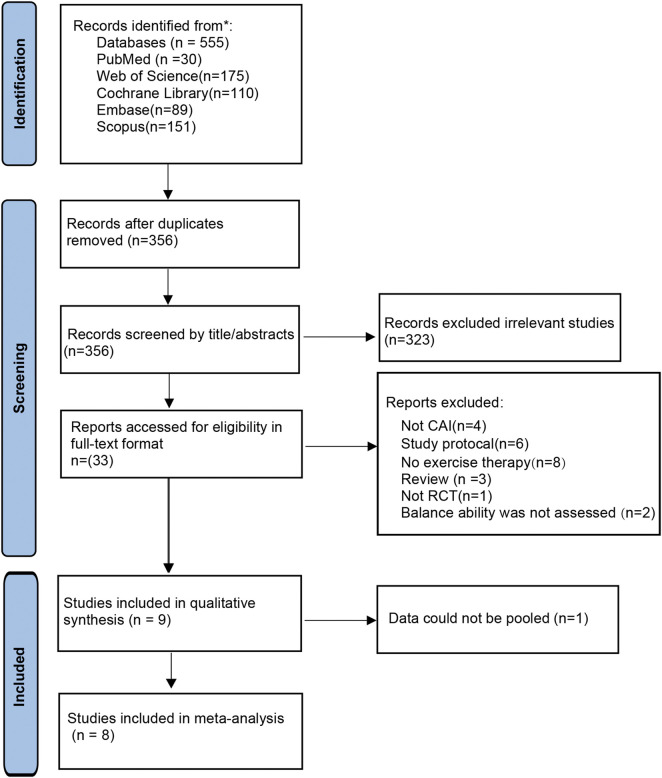
Flowchart of study screening.

The initial search across five databases generated 555 studies, which was reduced to 356 studies after removing duplicates. Following the application of inclusion and exclusion criteria through title and abstract screening, followed by full-text review, 9 studies were included for qualitative analysis (Bruce et al., 2020; Ma et al., 2020; Beyraghi and Khanmohammadi, 2025; Beyraghi et al., 2025; Ge et al., 2025; Kim et al., 2025; Needle et al., 2025; Sánchez-Barbadora et al., 2025; Zhang et al., 2025b). One study was excluded from quantitative synthesis due to incompatible data (Beyraghi et al., 2025), resulting in 8 studies being included in the meta-analysis (Bruce et al., 2020; Ma et al., 2020; Beyraghi and Khanmohammadi, 2025; Ge et al., 2025; Kim et al., 2025; Needle et al., 2025; Sánchez-Barbadora et al., 2025; Zhang et al., 2025b). The detailed selection process is illustrated in Figure 1.

### 3.2 Characteristics of the included studies

Among the included studies, a total of 248 individuals with CAI were included. The exercise therapy varied across studies: six studies implemented balance training (Beyraghi and Khanmohammadi, 2025; Beyraghi et al., 2025; Ge et al., 2025; Needle et al., 2025; Sánchez-Barbadora et al., 2025; Zhang et al., 2025b), one focused on short foot exercises (Ma et al., 2020), one utilized active joint mobilization techniques (Kim et al., 2025), and one employed ankle strengthening exercises (Bruce et al., 2020). Regarding control group interventions, two studies used exercise therapy alone (Kim et al., 2025; Sánchez-Barbadora et al., 2025), while the remaining seven studies employed exercise therapy combined with sham tDCS (Bruce et al., 2020; Ma et al., 2020; Beyraghi and Khanmohammadi, 2025; Beyraghi et al., 2025; Ge et al., 2025; Needle et al., 2025; Zhang et al., 2025b). For outcome measures, seven studies assessed dynamic balance ability (Bruce et al., 2020; Ma et al., 2020; Beyraghi and Khanmohammadi, 2025; Kim et al., 2025; Needle et al., 2025; Sánchez-Barbadora et al., 2025; Zhang et al., 2025b), with five utilizing the YBT (Ma et al., 2020; Beyraghi and Khanmohammadi, 2025; Kim et al., 2025; Sánchez-Barbadora et al., 2025; Zhang et al., 2025b) and two employing the DPSI (Bruce et al., 2020; Needle et al., 2025). Two studies evaluated static balance using COP sway measures (Ge et al., 2025; Kim et al., 2025), while one study evaluated static balance ability using the BESS (Zhang et al., 2025b), and one study assessed postural control during gait using COP sway amplitude (Beyraghi et al., 2025) (Table 1).

**TABLE 1:** Basic characteristics for include studies.

Study	Country	People (IG:CG) female proportion (IG: CG)	Age (IG:CG)	Experimental group	Control group	Main outcome measures	Time points of assessment
Sánchez-Barbadora et al. (2025)	Spain	10(IG):10(CG) 4/10(IG):3/10(CG)	21.7 ± 2.7(IG) 22.4 ± 1.9(CG)	tDCS + BB	BB	YBT composite reach distanceYBT_ANT/PM/PL	Baseline, Week 2, Week 4
Beyraghi et al. (2025)	Iran	15(IG):15(CG) 4/15(IG):5/15(CG)	27.47 ± 6.57(IG) 28.41 ± 5.58(CG)	tDCS + BT	sham tDCS + BT	YBT_ANT/PM/PL	Baseline, Week 2, Week 4
Beyraghi et al. (2025)	Iran	16(IG):16(CG) 4/16(IG):5/16(CG)	27.59 ± 6.45(IG) 27.46 ± 5.54(CG)	tDCS + BT	sham tDCS + BT	The displacement and velocity of the COP	Baseline, Week 4
Bruce et al. (2020)	USA	13(IG):13(CG) 10/13(IG):7/13(CG)	22.2 ± 2.8(IG) 22.5 ± 3.2(CG)	tDCS + ankle strength training	sham tDCS + ankle strength training	DPSI	Baseline, Week 2, Week 4, Week 6
Ge et al. (2025)	China	20(IG):20(CG) Gender not mentioned	20.1 ± 1.3(IG) 21.0 ± 1.8(CG)	HD-tDCS + BBT	sham HD-tDCS + BBT	COP_RMS	Baseline, Week 7
Kim et al. (2025)	Korea	12(IG):12(CG) 7/12(IG):6/12(CG)	20.33 ± 1.23(IG) 20.91 ± 1.72(CG)	tDCS + AJM	AJM	YBT composite reach distance The displacement of the CoP	Baseline, Week 4, Week 8
Ma et al. (2020)	China	14(IG):14(CG) 7/14(IG):8/14(CG)	21.14 ± 2.82(IG) 20.29 ± 1.49(CG)	HD-tDCS + SFE	sham HD-tDCS + SFE	YBT composite reach distance	Baseline, Week 1–6
Needle et al. (2025)	USA	15(IG):15(CG) 12/15(IG):11/15(CG)	22.6 ± 2.7(IG) 25.2 ± 8.7(CG)	tDCS + exercise	sham tDCS + exercise	DPSI	Baseline, Week 2, Week 4, Week 6
Zhang et al. (2025a)	China	9(IG):9(CG) 4/9(IG):4/9(CG)	20.00 ± 1.00(IG) 19.78 ± 0.97(CG)	tDCS + BT	sham tDCS + BT	YBT composite reach distanceYBT_ANT/PM/PL BESS	Baseline, Week 4

AJM, active joint mobilization; ANT, anterior reach; BB, black board instability device; BBT, bosu ball training; BESS, balance error scoring system BT, balance training; CG, control group; COP, center of pressure; DPS, dynamic postural stability indices; IG, Intervention group; PL, posterolateral reach; PM, posteromedial reach; SFE, short-foot exercise; YBT, Y-balance test

Concerning tDCS parameters, two studies utilized transcranial direct current stimulation (HD-tDCS) (Ma et al., 2020; Ge et al., 2025), five studies positioned the anode at Cz (Ma et al., 2020; Beyraghi and Khanmohammadi, 2025; Beyraghi et al., 2025; Ge et al., 2025; Zhang et al., 2025b), while others placed it at C3 or C4 (Bruce et al., 2020; Kim et al., 2025; Needle et al., 2025; Sánchez-Barbadora et al., 2025). Stimulation intensity ranged from 1.5 to 2 mA, with session durations of 10–20 min. One study conducted interventions for 6 weeks (Ge et al., 2025), while all others implemented 4-week intervention periods (Bruce et al., 2020; Ma et al., 2020; Beyraghi and Khanmohammadi, 2025; Beyraghi et al., 2025; Kim et al., 2025; Needle et al., 2025; Sánchez-Barbadora et al., 2025; Zhang et al., 2025b). For sham stimulation protocols, five studies applied current only during the initial 30 s (Ma et al., 2020; Beyraghi and Khanmohammadi, 2025; Beyraghi et al., 2025; Ge et al., 2025; Zhang et al., 2025b), one study used 2 min of initial stimulation (Bruce et al., 2020), and one employed 1 min of initial current application (Needle et al., 2025). Regarding adverse effects, two studies explicitly reported no adverse events during interventions (Needle et al., 2025; Zhang et al., 2025b), three studies indicated only mild and transient side effects (Bruce et al., 2020; Ma et al., 2020; Beyraghi et al., 2025), while four studies did not mention adverse effects (Beyraghi and Khanmohammadi, 2025; Ge et al., 2025; Kim et al., 2025; Sánchez-Barbadora et al., 2025) (Table 2).

**TABLE 2:** Parameters of tDCS.

Study	Site of anodal stimulation	Site of cathodal stimulation	Intensity of stimulation (mA)	Duration of stimulation (min)	Intervention frequency	Stimulation of sham tDCS	Adverse effects
Sánchez-Barbadora et al. (2025)	C3/C4	SO on the opposite side of the anode	2	10	3 times per week for 4 weeks	N/A	Not mentioned
Beyraghi and Khanmohammadi (2025)	Cz	Centrally on the forehead	1.5	20	3 times per week for 4 weeks	Stimulation with 2 mA current for the first 30 s	Not mentioned
Beyraghi et al. (2025)	Cz	Centrally on the forehead	1.5	20	3 times per week for 4 weeks	Stimulation with 2 mA current for the first 30 s	Mild, transient adverse effects
Bruce et al. (2020)	C3/C4	SO on the opposite side of the anode	1.5	18	5 times every 2 weeks for 4 weeks	Stimulation with 1.5 mA current for the first 2 min	Mild, transient adverse effects
Ge et al. (2025)	Cz	Fz、C3、Pz、C4	2	20	3 times per week for 6 weeks	Stimulation with 2 mA current for the first 30 s	Not mentioned
Kim et al. (2025)	C3/C4	SO on the opposite side of the anode	2	15	3 times per week for 4 weeks	N/A	Not mentioned
Ma et al. (2020)	Cz	Fz、C3、Pz、C4	2	20	3 times per week for 4 weeks	Stimulation with 2 mA current for the first 30 s	Mild, transient adverse effects
Needle et al. (2025)	C3/C4	SO on the opposite side of the anode	1.8	18	2 times per week for 4 weeks	Stimulation with 1.8 mA current for the first 1 min	No adverse effects were observed
Zhang et al. (2025a)	Cz	Fp2	2	20	3 times per week for 4 weeks	Stimulation with 2 mA current for the first 30 s	No adverse effects were observed

N/A, not applicable; SO, supraorbital area.

### 3.3 Risk of bias assessment

**FIGURE 2:**
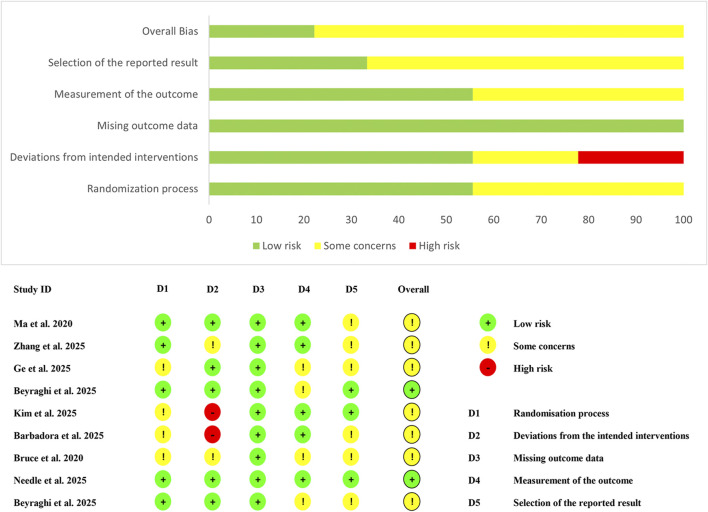
Risk of bias summary and graph.

Figure 2 shows the results of the risk of bias assessment for the included studies. For the randomization process domain, four studies failed to adequately describe their random allocation procedures and were rated as “some concerns” (Bruce et al., 2020; Ge et al., 2025; Kim et al., 2025; Sánchez-Barbadora et al., 2025), while the remaining studies demonstrated low risk. Regarding deviations from intended interventions, two studies were classified as high risk due to lack of blinding for both participants and intervention providers (Kim et al., 2025; Sánchez-Barbadora et al., 2025), and two additional studies received “some concerns” ratings for inadequate blinding of intervention providers (Bruce et al., 2020; Zhang et al., 2025b). All studies demonstrated low risk for missing outcome data. In the measurement of outcomes domain, four studies did not specify whether outcome assessors were blinded; however, considering the minimal influence of assessor knowledge on outcome measurement, these were rated as “some concerns” (Bruce et al., 2020; Beyraghi and Khanmohammadi, 2025; Beyraghi et al., 2025; Ge et al., 2025). For selective reporting, only three studies provided trial registration numbers and protocols (Beyraghi and Khanmohammadi, 2025; Kim et al., 2025; Needle et al., 2025), earning low risk ratings, while the remaining six studies were classified as “some concerns” due to insufficient reporting transparency (Bruce et al., 2020; Ma et al., 2020; Beyraghi et al., 2025; Ge et al., 2025; Sánchez-Barbadora et al., 2025; Zhang et al., 2025b).

Overall, merely two studies achieved an overall low risk of bias rating (Beyraghi and Khanmohammadi, 2025; Needle et al., 2025), with all other studies demonstrating “some concerns” across multiple domains (Bruce et al., 2020; Ma et al., 2020; Beyraghi et al., 2025; Ge et al., 2025; Kim et al., 2025; Sánchez-Barbadora et al., 2025; Zhang et al., 2025b), indicating moderate methodological limitations that may potentially influence the reliability of findings.

### 3.4 Meta-analysis results

Due to the limited number of studies and the diversity of balance assessment methods, dynamic balance ability was accessed by YBT composite score and the DPSI. In addition, subgroup analyses of the three YBT directional reach distances were conducted to assess the direction-specific characteristics of dynamic balance. For static balance, COP displacement and the BESS total score were pooled for analysis.

#### 3.4.1 Effects of combined exercise therapy and tDCS on dynamic balance

**FIGURE 3:**
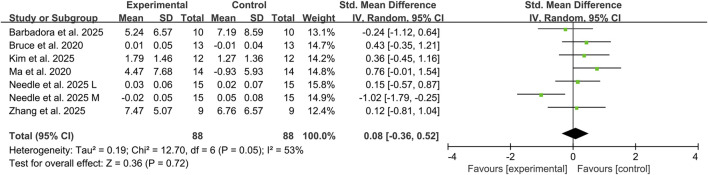
Effects of combined exercise therapy and tDCS on dynamic balance. Note: L, lateral; M, medial.

Six studies assessed the impact of combined tDCS and exercise therapy on dynamic balance performance in CAI individuals (Bruce et al., 2020; Ma et al., 2020; Kim et al., 2025; Needle et al., 2025; Sánchez-Barbadora et al., 2025; Zhang et al., 2025b), utilizing YBT composite scores and DPSI measurements across 146 CAI participants. Meta-analysis results showed that compared with exercise therapy alone, the addition of tDCS did not significantly improve dynamic balance ability in CAI individuals (SMD: 0.08, 95% CI: −0.36 to 0.52; *P* = 0.72), representing a trivial effect size, with substantial heterogeneity observed between studies (I^2^ = 53%) (Figure 3).

#### 3.4.2 Subgroup analysis of combined exercise therapy and tDCS on dynamic balance

##### 3.4.2.1 Different type of exercise therapy

**FIGURE 4:**
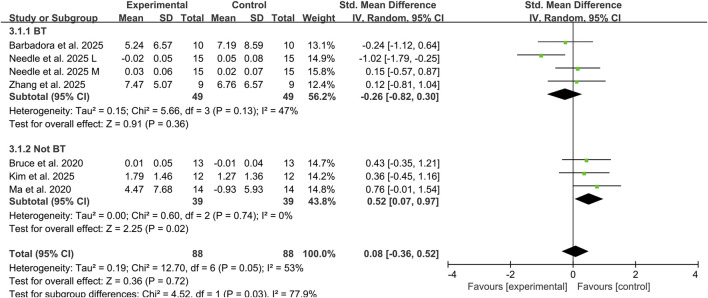
Subgroup analysis of the effects of tDCS combined with different types of exercise therapy on dynamic balance ability. Note: BT, balance training; L, lateral; M, medial.

Three studies assessed the effects of tDCS combined with balance training on dynamic balance ability in CAI individuals using YBT composite scores and DPSI, involving 68 participants (Needle et al., 2025; Sánchez-Barbadora et al., 2025; Zhang et al., 2025b). The addition of tDCS did not significantly improve dynamic balance ability, showing a small effect size (SMD: −0.26, 95% CI: −0.82 to 0.30, *P* = 0.36), with moderate heterogeneity between studies (I^2^ = 47%). Another three studies evaluated the effects of combined non-balance training on dynamic balance ability, involving 58 participants (Bruce et al., 2020; Ma et al., 2020; Kim et al., 2025). The results demonstrated a medium effect size in favor of combined tDCS and non-balance training for improving dynamic balance ability in CAI individuals, (SMD: 0.52, 95% CI: 0.07 to 0.97, *P* = 0.02) and no heterogeneity observed between studies (I^2^ = 0%) (Figure 4).However, these findings should be interpreted with caution given the limited number of studies in this subgroup analysis.

##### 3.4.2.2 Different directions of YBT

**FIGURE 5:**
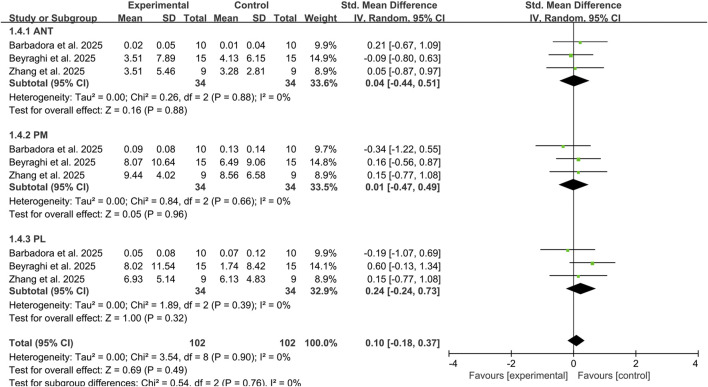
Subgroup analysis of the effects of tDCS combined with exercise therapy on each direction of the YBT. Note: ANT, anterior reach; PL, posterolateral reach; PM, posteromedial reach.

Three studies assessed the impact of combined tDCS and exercise therapy on YBT reach distances in three directions among 68 CAI participants (Beyraghi and Khanmohammadi, 2025; Sánchez-Barbadora et al., 2025; Zhang et al., 2025b). The results revealed trivial to small effect sizes with no significant improvements in any direction: ANT (SMD: 0.04, 95% CI: −0.44 to 0.51, *P* = 0.88), PM (SMD: 0.01, 95% CI: −0.47 to 0.49, *P* = 0.96), PL (SMD: 0.24, 95% CI: −0.24 to 0.73, *P* = 0.32). No heterogeneity was observed between studies across all three directions (I^2^ = 0%) (Figure 5). These directional analyses should be interpreted cautiously due to the limited evidence base.

#### 3.4.3 Effects of combined exercise therapy and tDCS on static balance

**FIGURE 6:**
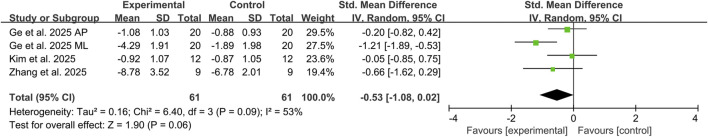
Effects of combined exercise therapy and tDCS on static balance. Note: AP, anteroposterior; ML, mediolateral.

Three studies examined the effects of combined tDCS and exercise therapy on static balance in CAI, with two studies using COP (Ge et al., 2025; Kim et al., 2025) and one study using BESS for assessment (Zhang et al., 2025b), involving a total of 82 participants. Figure 6 showed that combined tDCS and exercise therapy failed to show significant enhancement in static balance performance among CAI, demonstrating a medium effect size (SMD: −0.53, 95% CI: −1.08 to 0.02, *P* = 0.06), with substantial heterogeneity observed between studies (I^2^ = 53%) (Figure 6). Given the limited number of studies, this finding require cautious interpretation.

### 3.5 Sensitivity analysis results

To identify potential sources of high heterogeneity, sensitivity analyses were performed. Exclusion of the study by Needle et al. reduced heterogeneity for dynamic balance ability from I^2^ = 53%–0%, and in the tDCS combined with balance training subgroup from I^2^ = 47%–0%. For static balance outcomes, exclusion of the medial-lateral COP values reported by Ge et al. reduced heterogeneity from I^2^ = 53%–0%. To assess the stability of results, removal of the study by Ma et al. rendered the subgroup analysis of tDCS combined with non-balance training on dynamic balance ability non-significant (P = 0.17). Conversely, exclusion of the study by Kim et al. resulted in a significant pooled effect for static balance (P = 0.04). The other analyses were unaffected by individual study. Details are presented in Supplementary Table S2.

These results indicate that Needle et al. and Ge et al. were the primary sources of heterogeneity for dynamic and static balance outcomes, respectively. Importantly, the pooled effects for static balance and for tDCS combined with non-balance training on dynamic balance demonstrated instability, being significantly influenced by individual studies.

### 3.6 Certainty of evidence

**FIGURE 7:**
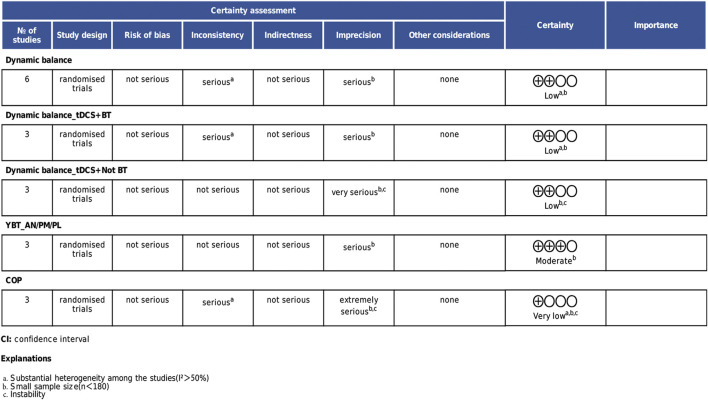
Certainty of evidence.

According to the GRADE assessment, the quality of evidence varied across outcomes. The overall dynamic balance results and the subgroup analysis of dynamic balance combined with balance training were rated as low quality due to high heterogeneity and insufficient sample sizes. Static balance results were rated as very low quality due to result instability. Within the dynamic balance subgroup analyses, the YBT composite score analysis was rated as moderate quality due to limited sample size, while the subgroup combined with non-balance training was downgraded to low quality due to result instability. Details are presented in Figure 7.

## 4 Discussion

This systematic review and meta-analysis provides the first comprehensive evaluation of combined tDCS and exercise therapy effects on balance performance in CAI individuals. The results show that when tDCS is combined with non-balance training, it provides additional improvements in dynamic balance ability. However, when combined with balance training, no significant additional benefits are observed. Regarding static balance, the combination of tDCS with exercise therapy showed no significant improvement compared to exercise therapy alone.

### 4.1 Dynamic balance

Balance dysfunction is common in CAI individuals, potentially resulting from central nervous system reorganization following ligament injury (Thompson et al., 2018; Ghislieri et al., 2023). CAI individuals exhibit reduced cortical excitability in the primary motor cortex (M1) and supplementary motor area (SMA), which disrupts motor control and leads to balance deficits (Solis-Escalante et al., 2019; Lepley and Lepley, 2022; Egger et al., 2023). Since tDCS can enhance M1 cortical excitability through anodal stimulation and improve motor learning and balance control (Devanathan and Madhavan, 2016; Kaminski et al., 2016), combining tDCS with exercise therapy should theoretically provide synergistic benefits for addressing dynamic balance deficits in CAI individuals. However, our meta-analysis shows that tDCS provides additional clinical benefits only when combined with non-balance training (strength training, SFE, and AJM). When tDCS combined with balance training, neither the comprehensive dynamic balance assessment indicators (YBT composite scores and DPSI) nor the specific directional results of YBT testing showed significant differences compared to balance training alone. This finding may have important clinical and scientific implications. It suggests that the effectiveness of tDCS as an adjunctive treatment for improving dynamic balance in CAI may have conditional limitations, potentially related to differences in neural networks and plasticity mechanisms activated by different training modalities.

From a neurophysiological perspective, a possible hypothesis may explain why the combination of tDCS and balance training failed to yield additional benefits. Balance training primarily enhances the efficacy of GABAergic inhibitory pathways in the motor cortex, significantly increasing short-interval intracortical inhibition (SICI), thereby suppressing co-contraction of irrelevant muscles and achieving refined motor control regulation (Mouthon and Taube, 2019; Taube et al., 2020). In contrast, strength training reduces SICI to release M1 output potential and achieve increased strength (Kidgell et al., 2017). However, existing meta-analyses have confirmed that anodal tDCS suppresses GABA synthesis and weakens SICI function (Biabani et al., 2018). This contradiction in neuroplasticity mechanisms may be one of reasons why tDCS combined with balance training fails to further improve balance capacity in CAI individuals. However, we must cautiously note that this explanation remains speculative at present. First, no relevant studies have validated the baseline characteristics of SICI in CAI populations, which makes it unclear whether interventions aimed at modulating cortical inhibition are equally applicable in individuals with CAI. Second, none of the studies included in this meta-analysis directly measured neurophysiological indicators such as SICI through techniques like TMS to confirm that tDCS and balance training indeed induced the aforementioned mutually antagonistic neuromodulatory effects in participants’ brains. Therefore, this hypothesis still requires further validation through future research. Furthermore, balance training alone has been extensively demonstrated to significantly improve dynamic balance function in CAI individuals, and the resulting ceiling effect may also limit the potential for additional benefits from tDCS (Mollà-Casanova et al., 2021; Guo et al., 2024).

It should also be noted that although our findings indicate that tDCS combined with different types of exercise therapy may exert differential effects on dynamic balance with CAI, the limited number of studies included in the subgroup analyses and the instability of the pooled results for tDCS combined with balance training in the sensitivity analysis suggest that these conclusions should be interpreted with caution. Further high-quality randomized controlled trials are warranted to substantiate these findings.

### 4.2 Static balance

This meta-analysis shows that adding tDCS to exercise therapy did not significantly enhance static balance ability in CAI individuals compared to exercise therapy alone. Neuroimaging studies reveal that dynamic balance tasks elicit higher activation in motor control networks, requiring elevated cortical excitability to coordinate complex muscle activation patterns and postural adjustments (Taube et al., 2015). In contrast, static balance control relies on automated postural mechanisms with lower neural resource demands (Jahn et al., 2004). Consequently, the neuromodulatory effects of tDCS through enhanced cortical excitability may have limited impact on improving static balance control (Xiao et al., 2020; Xiao et al., 2022). However, several limitations should be acknowledged. Subgroup analyses examining different exercise therapy types could not be conducted for static balance outcomes due to the limited study sample, which prevented assessment of whether specific training modalities demonstrate superior synergistic effects with tDCS. Additionally, the included studies exhibited heterogeneity in static balance assessment methods, which may have affected the accuracy of the meta-analysis results.

Although the overall results did not reach statistical significance, the effect size and confidence intervals suggest that tDCS may have potential clinical benefits in improving static balance. Furthermore, sensitivity analysis revealed that the results became statistically significant after excluding the Kim et al. Combined with findings from Ge et al. which demonstrated that tDCS combined with exercise therapy significantly improved mediolateral sway amplitude of the COP in individuals with CAI, these findings indicate that tDCS combined with exercise therapy may indeed improve static balance in CAI. However, this effect is currently obscured by between-study heterogeneity and insufficient sample sizes. Future research with larger sample sizes and more rigorous designs is needed to validate and elucidate this potential therapeutic effect.

### 4.3 Clinical implications

Our research findings provide certain guidance for the clinical application of CAI rehabilitation. In clinical practice, individualized treatment protocols should be developed based on specific patient conditions. For CAI individuals who have not received balance training, tDCS can serve as an effective adjunctive therapy to significantly improve their dynamic balance ability. However, for individuals already undergoing systematic balance training, the additional use of tDCS may not provide extra benefits for balance function improvement. Regarding stimulation parameters, based on current evidence, we recommend adopting a treatment protocol of 1.5–2 mA anodal stimulation over the M1 area for 15–20 min. Nevertheless, due to the limited number of related studies, future high-quality research is still needed to explore the effects of different stimulation intensities, durations, and target sites on balance function in CAI individuals, thereby establishing optimal stimulation parameters to provide more precise evidence for clinical decision-making.

### 4.4 Limitations

This study has several limitations: 1. The relatively small sample sizes of included studies and heterogeneity observed in some meta-analyses resulted in low to moderate certainty of evidence; 2. Variations in balance assessment methods across studies, while not identified as a major source of heterogeneity in sensitivity analysis, may still affect the precision of results; 3. Limited by the insufficient number of studies on static balance, subgroup analyses by different exercise therapy types were not feasible, preventing clear determination of differential effects of tDCS combined with various exercise interventions on static balance; 4. Analyses stratified by tDCS stimulation parameters and intervention duration were not conducted, leaving unclear whether different stimulation parameters and treatment durations produce varying effects.

## 5 Conclusion

Our findings demonstrate that the effectiveness of tDCS combined with different types of exercise therapy on dynamic balance improvement in individuals with CAI may vary. Preliminary evidence indicates that tDCS combined with non-balance training may offer potential benefits for dynamic balance ability, though this finding requires further validation due to its demonstrated instability. Conversely, tDCS combined with balance training showed no significant additional benefits. Regarding static balance ability, current evidence is insufficient to demonstrate that tDCS combined with exercise therapy provides further improvement effects. Future research should conduct more high-quality randomized controlled trials to validate this conclusion and systematically explore optimal combination protocols between different exercise therapy modalities and tDCS, while establishing ideal stimulation parameter configurations, thereby providing higher-quality evidence-based medicine for clinical practice.

## Data Availability

The original contributions presented in the study are included in the article/Supplementary Material, further inquiries can be directed to the corresponding author.
