# Supplementary material for: Can exercise therapy combined with transcranial direct current stimulation further improve balance ability in individuals with chronic ankle instability? A systematic review and meta-analysis
Source: Front Physiol. 2025 Oct 15;16:1681272. doi: 10.3389/fphys.2025.1681272 (PMC12568559; doi:10.3389/fphys.2025.1681272)
Supplement: Supplementary file 1 [file Table1.docx]

***Search Strategy***

**Databases:**

**PubMed**: 30 results

#1: ((((("Transcranial Direct Current Stimulation"[Mesh]) OR (Transcranial Direct Current Stimulation[Title/Abstract])) OR (tDCS[Title/Abstract])) OR (Transcranial Electrical Stimulation[Title/Abstract])) OR (Cathodal Stimulation[Title/Abstract])) OR (anodal Stimulation[Title/Abstract])

#2: (((ankle[MeSH Terms]) OR (ankle instability[Title/Abstract])) OR (chronic ankle instability[Title/Abstract])) OR (funcational ankle instability[Title/Abstract])

Final: #1 AND #2

**Web of sciences**: 175 results

#1: TS=( transcranial direct current stimulation OR tDCS OR Transcranial Electrical Stimulation OR Cathodal Stimulation OR anodal Stimulation)

#2: TS=( ankle* OR ankle instability OR chronic ankle instability OR Functional Ankle Instability)

Final: #1 AND #2

**Embase**: 89 results

#1: 'ankle':ab,ti OR 'ankle instability':ab,ti OR 'chronic ankle instability':ab,ti OR 'functional ankle instability'::ab,ti

#2: 'transcranial direct current stimulation':ab,ti OR tDCS:ab,ti OR 'Transcranial Electrical Stimulation':ab,ti OR 'Cathodal Stimulation':ab,ti OR 'anodal Stimulation':ab,ti

Final: #1 AND #2

**Scopus:** 151 results

( TITLE-ABS-KEY ( ankle ) OR TITLE-ABS-KEY ( ankle instability ) OR TITLE-ABS-KEY ( chronic ankle instability ) OR TITLE-ABS-KEY ( functional ankle instability ) ) AND ( TITLE-ABS-KEY ( transcranial direct current ) OR TITLE-ABS-KEY ( tDCS ) OR TITLE-ABS-KEY ( Transcranial Electrical Stimulation ) OR TITLE-ABS-KEY ( Cathodal Stimulation ) OR TITLE-ABS-KEY ( anodal Stimulation ) )

**Cochrane**: 110 results

#1: (transcranial direct current stimulation):ti,ab,kw OR (tDCS):ti,ab,kw OR (Transcranial Electrical Stimulation):ti,ab,kw OR (Cathodal Stimulation):ti,ab,kw OR (anodal Stimulation):ti,ab,kw

#2: (ankle):ti,ab,kw OR (ankle instability):ti,ab,kw OR (chronic ankle instability):ti,ab,kw OR (functional ankle instability):ti,ab,kw

Final: #1 AND #2
