# Supplementary material for: Can exercise therapy combined with transcranial direct current stimulation further improve balance ability in individuals with chronic ankle instability? A systematic review and meta-analysis
Source: Front Physiol. 2025 Oct 15;16:1681272. doi: 10.3389/fphys.2025.1681272 (PMC12568559; doi:10.3389/fphys.2025.1681272)
Supplement: Supplementary file 2 [file Table2.docx]

| Meta-analysis results | Subgroup | Excluded study | I²(%) | Analysis results | |
| --- | --- | --- | --- | --- | --- |
|  |  |  |  | SMD, 95%CI | *P* |
| Effects of combined exercise therapy and tDCS on dynamic balance. |  | Barbadora et al. 2025 | 59 | 0.13 [-0.37, 0.63] | 0.61 |
|  |  | Bruce et al. 2020 | 58 | 0.02 [-0.49, 0.53] | 0.94 |
|  |  | Kim et al. 2025 | 59 | 0.03 [-0.48, 0.55] | 0.89 |
|  |  | Ma et al. 2020 | 45 | -0.04 [-0.49, 0.41] | 0.87 |
|  |  | Needle et al. 2025 L | 0 | 0.29 [-0.04, 0.61] | 0.09 |
|  |  | Needle et al. 2025 M | 61 | 0.07 [-0.46, 0.60] | 0.80 |
|  |  | Zhang et al. 2025 | 61 | 0.08 [-0.44, 0.59] | 0.77 |
|  |  |  |  |  |  |
| Subgroup analysis of the effects of tDCS combined with different types of exercise therapy on dynamic balance ability | BT | Barbadora et al. 2025 | 65 | -0.26 [-1.04, 0.51] | 0.51 |
|  |  | Needle et al. 2025 L | 0 | 0.03 [-0.45, 0.50] | 0.92 |
|  |  | Needle et al. 2025 M | 47 | -0.42 [-1.10, 0.25] | 0.22 |
|  |  | Zhang et al. 2025 | 59 | -0.37 [-1.07, 0.34] | 0.31 |
|  |  |  |  |  |  |
|  | Not BT | Bruce et al. 2020 | 0 | 0.57 [0.01, 1.13] | 0.05 |
|  |  | Kim et al. 2025 | 0 | 0.60 [0.05, 1.15] | 0.03 |
|  |  | Ma et al. 2020 | 0 | 0.39 [-0.17, 0.95] | 0.17 |
|  |  |  |  |  |  |
| Subgroup analysis of the effects of tDCS combined with exercise therapy on each direction of the YBT | ANY | Barbadora et al. 2025 | 0 | -0.03 [-0.60, 0.53] | 0.91 |
|  |  | Beyraghi et al. 2025 | 0 | 0.13 [-0.50, 0.77] | 0.68 |
|  |  | Zhang et al. 2025 | 0 | 0.03 [-0.52, 0.59] | 0.91 |
|  |  |  |  |  |  |
|  | PM | Barbadora et al. 2025 | 0 | 0.15 [-0.41, 0.72] | 0.59 |
|  |  | Beyraghi et al. 2025 | 0 | -0.10 [-0.74, 0.54] | 0.75 |
|  |  | Zhang et al. 2025 | 0 | -0.04 [-0.60, 0.52] | 0.89 |
|  |  |  |  |  |  |
|  | PL | Barbadora et al. 2025 | 0 | 0.43 [-0.14, 1.01] | 0.14 |
|  |  | Beyraghi et al. 2025 | 0 | -0.03 [-0.66, 0.61] | 0.94 |
|  |  | Zhang et al. 2025 | 0 | 0.25 [-0.53, 1.02] | 0.53 |
|  |  |  |  |  |  |
| Effects of combined exercise therapy and tDCS on static balance. |  | Ge et al. 2025 AP | 58 | -0.66 [-1.38, 0.05] | 0.07 |
|  |  | Ge et al. 2025 ML | 0 | -0.25 [-0.69, 0.19] | 0.26 |
|  |  | Kim et al. 2025 | 57 | -0.68 [-1.33, -0.03] | 0.04 |
|  |  | Zhang et al. 2025 | 68 | -0.50 [-1.21, 0.22] | 0.71 |
| AP, anteroposterior; ANY, anterior reach; BT, balance training; CI, confidence intervals; L, lateral; M, medial; ML, mediolateral; PM, posteromedial reach; PL, posterolateral reach; SMD, standardized mean differences; tDCS, transcranial direct current stimulation | | | | | |
